# Estimating residual undifferentiated cells in human chemically induced pluripotent stem cell derived islets using lncRNA as biomarkers

**DOI:** 10.1038/s41598-023-43798-0

**Published:** 2023-09-30

**Authors:** Yandan Wu, Zhenzhen Zhang, Shuangshuang Wu, Zhaolong Chen, Yue Pu

**Affiliations:** Hangzhou Reprogenix Bioscience Co., Ltd, Hangzhou, 310023 China

**Keywords:** Induced pluripotent stem cells, Long non-coding RNAs

## Abstract

Human pluripotent stem cells (hPSCs) can generate insulin-producing beta cells for diabetes treatment, but residual undifferentiated cells may cause tumors. We developed a highly sensitive assay to detect these cells in islet cells derived from human chemically induced pluripotent stem cells (hCiPSCs), which are transgene-free and safer. We used RNA-seq data to find protein-coding and non-coding RNAs that were only expressed in hCiPSCs, not in islet cells. We confirmed these biomarkers by RT-qPCR and ddPCR. We chose long non-coding RNA (lncRNA) markers, which performed better than protein-coding RNA markers. We found that *LNCPRESS2, LINC00678* and *LOC105370482* could detect 1, 1 and 3 hCiPSCs in 10^6^ islet cells by ddPCR, respectively. We tested our method on several hCiPSC lines, which could quantify 0.0001% undifferentiated cell in 10^6^ islet cells by targeting hCiPSCs-specific lncRNA transcripts, ensuring the safety and quality of hCiPSC-derived islet cells for clinical use.

## Introduction

Type 1 diabetes (T1D) is a chronic condition that requires lifelong treatment with exogenous insulin injections or donor islet transplantation^1,2^. However, both options have limitations: insulin injections can cause hyperglycemia and hypoglycemia events, while donor islet transplantation faces the challenges of donor scarcity and immune rejection^3,4^. Therefore, there is a need for alternative sources of functional pancreatic islet cells that can restore glucose homeostasis in patients with T1D. One promising approach is to differentiate human pluripotent stem cells (hPSC) into pancreatic islet cells, inspired by the success of islet transplantation. Among the various types of hPSC, chemically induced pluripotent stem cells (CiPSC) are particularly attractive for clinical applications, as they are generated by a transgene-free method that uses chemical compounds to reprogram somatic cells into pluripotent state^5,6^. This method avoids the manipulation of tumorigenic genes such as c-Myc, which may increase the risk of cancer in other types of iPSC^7^. Moreover, recent studies have shown that islets derived from hCiPSC can ameliorate diabetes in non-human primates, suggesting their potential for islet replacement therapy^8,9^. However, before hCiPSC-derived islets can be used in humans, their safety must be ensured by eliminating the residual undifferentiated hCiPSCs in the final cell products, as they may form teratomas or malignant tumors. Therefore, it is critical to establish a specific and sensitive method for evaluating the purity and quality of hCiPSC-derived islets.

Teratoma formation is affected by several factors, such as the characteristics of the iPSCs, the transplantation location, the resuspension buffer, and the cell number. Among these factors, cell number is crucial because it determines the amount of iPSCs that can potentially form tumors. Previous studies have reported that a minimum of 1 × 10^5^ ES cells in the myocardium and 1 × 10^4^ cells in the skeletal muscle are required for teratoma development^10^. Another study has also shown that 2 × 10^5^ iPSCs are sufficient to induce teratomas^11^. In clinical settings, the dose level administered to patients may be as high as 10^9^–10^10^ cells according to non-human primates experiments^8^. Therefore, it is essential to develop ultra-sensitive assays that can detect as few as 10^4^ hCiPSCs in 10^10^ cells (0.0001%) to assess the teratoma risk from residual hCiPSCs.

Current techniques for detecting hiPSCs residues include in vivo and in vitro approaches. The in vivo teratoma testing, which involves injecting cells into mice with severe combined immunodeficiency (SCID) and observing tumor formation, is a direct method to assess the tumorigenicity^12^. Another in vivo method is positron emission tomography (PET) with [18F] FEDAC (a TSPO radioligand), which can monitor the remnant undifferentiated hiPSCs after hiPSC-derived neural stem/progenitor cells transplantation^13^. However, these methods are costly and time-consuming, which limit their applicability. The classical in vitro methods, such as the high-efficiency culture (HEC) system, use laminin-521 and Essential 8 medium to detect hiPSCs residual in primary human mesenchymal stem cells (hMSCs) with a sensitivity of 0.001–0.01%^14^. This sensitivity can be further improved by combining the HEC assay with magnetic-activated cell sorting (MACS), reaching a detection level of 0.00002%^15^. However, this method is also time-consuming and labor-intensive. Other in vitro methods, such as flow cytometry, use specific surface antigens of stem cells, such as TRA-1-60^16^, to identify hiPSCs residues. However, these methods may not be sensitive enough to detect low levels of hiPSCs and may be influenced by gating techniques. Some novel methods have been developed to detect or eliminate hiPSCs residues, but they are either complicated or not widely available. For example, glycosphingolipid-glycome analysis can detect iPSC-specific GSL-glycans from 5 × 10^4^ chondrocytes cells^17^, surface-enhanced Raman scattering (SERS)-based assay can identify as few as 1 stem cell in 10^6^ cells^18^, and cytotoxic antibodies can target and kill hPSCs residues^19,20^.

Several other methods based on PCR platform have been developed, such as RT-qPCR-based and ddPCR-based assays. These methods are convenient and rapid, as they can amplify and detect specific nucleic acid sequences. For example, Sekine K, et al. used RT-qPCR to detect common pluripotency markers such as *SRY-Box Transcription Factor 2 (SOX2)*, *Nanog Homeobox (NANOG)*, *Organic cation/carnitine transporter4 (OCT4, also known as POU5F1)* and *Lin-28 Homolog A (LIN28A)*, which can distinguish undifferentiated iPSCs from differentiated cells^21^. Liam Chung et al. used iPSC-specific microRNA as a target for detecting iPSCs in the background of iPSC-derived natural killer (iNK) cells using ddPCR^22^. However, these methods have two main limitations: (1) they may not have sufficient sensitivity and specificity for clinical applications; (2) the targets used were not verified of enough iPSC lines. To overcome these limitations, we screened lncRNA markers that were suitable for several hCiPSC lines and their derived islet cells, making universal markers.

Non-coding RNAs (ncRNAs) are essential regulators in biological processes, transcribed from most of the mammalian genome (> 90%) that was once deemed as ‘junk DNA’^23,24^. LncRNAs are a type of ncRNAs with sequences over 200 nucleotides^25^. They have lower conservation and expression than mRNA, but they play multifunctional roles in cells, including in stem cell self-renewal and differentiation^26^. For example, some lncRNAs are crucial for maintaining pluripotency and interacting with core transcription factors (TFs) OCT4, NANOG, and SOX2 in mouse embryonic stem cells (mESCs)^27^ and human embryonic stem cells (hESCs)^28^. Other lncRNAs promote cell reprogramming by inhibiting p53-mediated cell cycle arrest and apoptosis^29,30^, or act as competing endogenous RNAs (ceRNAs) that sequester miRNAs that repress core TFs^31,32^. These examples illustrate the diverse and complex functions of lncRNAs in regulating stem cell fate.

In this study, we developed a lncRNA detection assay to detect residual undifferentiated cells in hCiPSCs derived islets product. These lncRNAs were selected from differentially expressed RNA profile and are specific to hCiPSCs and their derived islet cells. By combining the sensitivity of the ddPCR platform, we demonstrate that this lncRNA detection assay could be effective across several hCiPSC lines and could detect as few as 1 undifferentiated hCiPSC in the background of 10^6^ hCiPSC-derived islet cells. This assay provides a research paradigm that might be promising for finding specific biomarkers and detecting residual undifferentiated cells in other hCiPSC derived cell products.

## Results

### RNA expression pattern and the candidate markers

**Figure 1:**
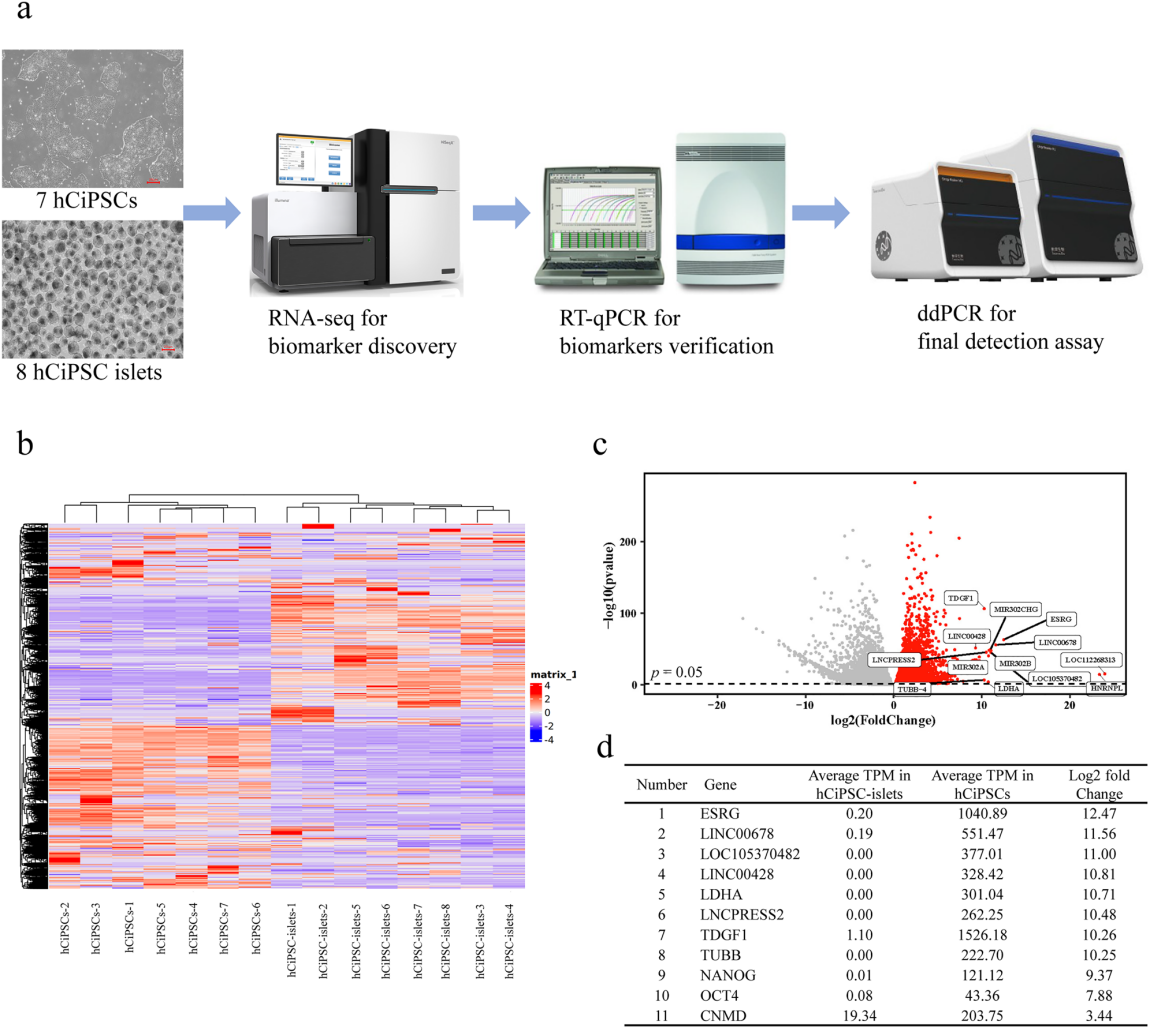
Research scheme and RNA-seq results. (**a**) Research scheme for discovery and verification of hCiPSC-islets specific biomarkers. (**b**) Heatmap and hierarchical clustering between seven hCiPSCs and eight hCiPSC-islets samples. Gene transcripts with *p* value < 0.05 were displayed, red indicates high expression and blue indicates low expression. (**c**) Volcano plot showing differential expression between hCiPSCs and hCiPSC-islets. Genes with *p* value < 0.05, log2 fold change > 10 were colored in red. (**d**) Average TPM of selected markers in hCiPSCs and hCiPSC-islets. TPM and log2 fold change were shown.

We followed a discovery and verification procedure to identify candidate marker genes that are highly expressed in hCiPSCs but not in differentiated cells, which would enable us to detect and quantify hCiPSC residue in hCiPSC-derived cell products (Fig. 1a). To do this, we sequenced and analyzed RNA expression patterns of seven hCiPSCs and eight hCiPSC-islets (Table S1). We used hierarchical clustering to compare the gene expression profiles of hCiPSCs and hCiPSC-islets and displayed all genes with *p* value < 0.05 in a heatmap, where red indicates high expression and blue indicates low expression (Fig. 1b). The heatmap shows that hCiPSCs and hCiPSC-islets have distinct gene expression patterns, but there were also variations within each group. This suggests that hCiPSCs and their derived cells are slightly different depending on the donors and differentiation respectively, which makes it more difficult to find specific markers and requires more validation for different hCiPSC lines and their differentiated islets. We selected 13 candidates with significant differences between the two cell types, based on *p* value < 0.05 and log2 fold change > 10 (Fig. 1c). We further filtered the candidates by requiring TPM > 200 in hCiPSCs, which demands promising markers should be highly expressed. We found eight candidate genes that met these criteria: *Embryonic Stem Cell Related (ESRG)*, *Long Intergenic Non-Protein Coding RNA 678 (LINC00678)*, *Uncharacterized LOC105370482*, *Long Intergenic Non-Protein Coding RNA 428 (LINC00428)*, *Lactate Dehydrogenase A (LDHA)*, *LncRNA P53 Regulated And ESC Associated 2 (LNCPRESS2)*, *Teratocarcinoma-Derived Growth Factor 1 (TDGF1),* and *Tubulin Beta Class I (TUBB)* (Fig. 1d). These genes belong to the RNA coding gene class and non-coding RNA gene class, indicating a complex pluripotency network in hCiPSCs. In addition, we also included biomarkers of *OCT4*, *NANOG* and *Chondromodulin (CNMD)* based on previous studies^21^. In summary, we selected 11 genes as candidate markers for further verification (Fig. 1d).

### Preliminary verification of candidate markers by RT-qPCR

**Figure 2:**
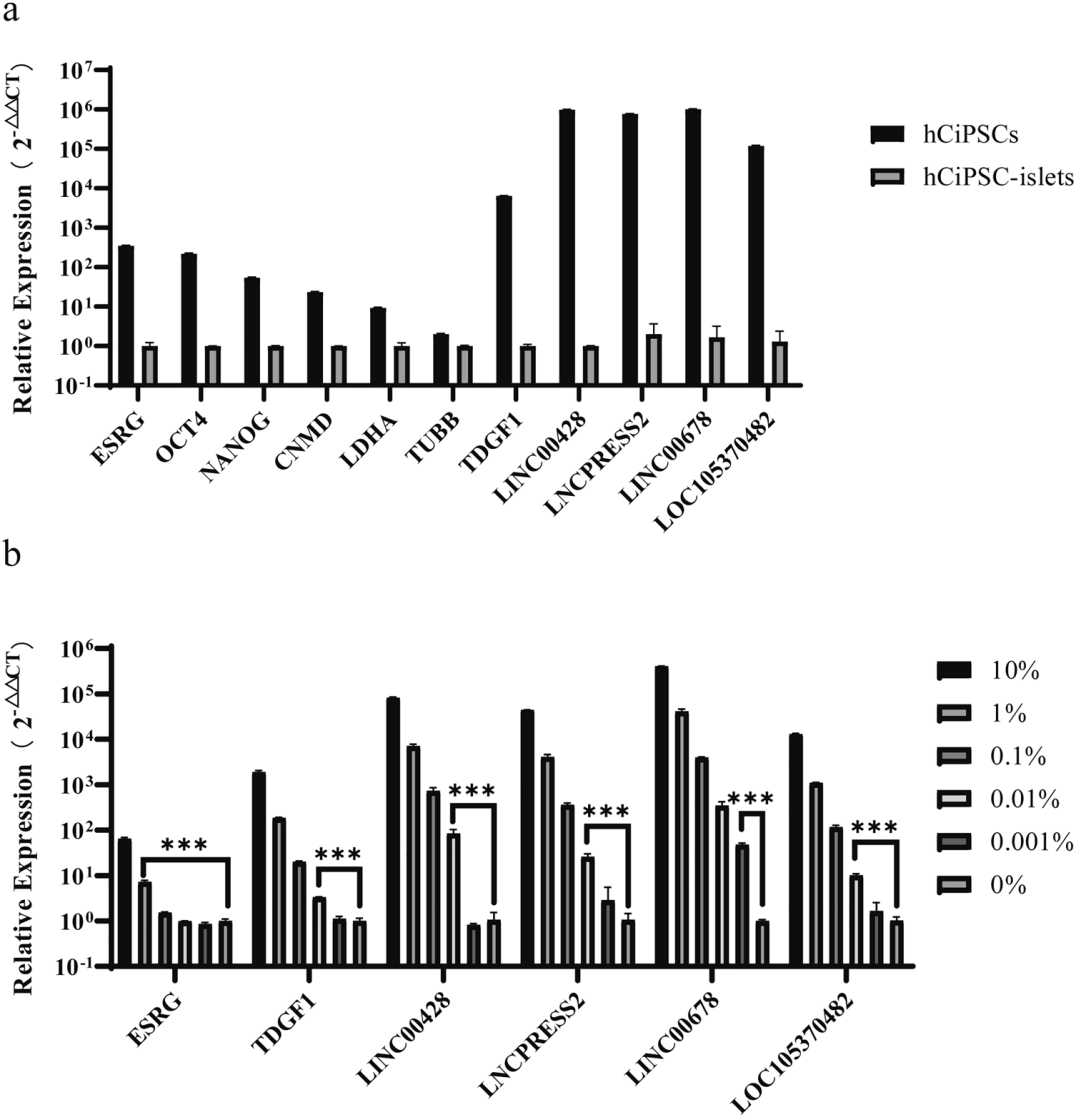
RT-qPCR detection results of candidate markers. (**a**) The qPCR results showing relative expression level of candidate markers in hCiPSCs compared with hCiPSC-islets. (**b**) Relative expression level of selected markers in RNA mixtures. Each marker had five mixing ratios and relative expression level of each RNA mixture compared with 0%. Results are presented as the mean ± standard deviation (n = 3). *** *p* < 0.001.

We tested the capability of these candidate markers for detecting residual undifferentiated cells in hCiPSC-derived islet cells by RT-qPCR assay. First, we measured the expression levels of 11 candidates in hCiPSCs and hCiPSC-islets by RT-qPCR, using the same amount of RNA input. The results showed that the selected markers had higher expression levels in hCiPSCs than in hCiPSC-islets, but their expression levels and fold changes varied (Fig. 2a). The non-coding RNAs *LINC00428*, *LNCPRESS2*, *LINC00678* and *LOC105370482* had the highest expression levels among all 11 candidates, while only *TDGF1*, a protein-coding gene, had a fold change > 1000. These results of RT-qPCR were not fully consistent with RNA-seq data, which might be influenced by the different methods used and the lower sequencing depth of RNA-seq. The markers selected from previous studies, such as *OCT4*, *NANOG* and *CNMD*, showed poor performance, indicating that specific markers were essential. Based on the criteria of high expression level and fold change in hCiPSCs compared with differentiated cells, we excluded genes like *LDHA*, *TUBB*, *OCT4*, *NANOG* and *CNMD* from further study.

Next, we performed an RNA spike-in study to measure the detection sensitivity of markers in RNA mixtures. The results showed that the relative RNA expression decreased with the decreasing amount of hCiPSCs RNA in samples. The detection capability varied for each candidate marker. *LINC00678* could detect a significant difference between 0.001% and 0% (p < 0.001), while *TDGF1*, *LINC00428*, *LNCPRESS2* and *LOC105370482* could detect 0.01% (p < 0.001), and *ESRG* only 0.1% (p < 0.001) (Fig. 2b). Based on their detection capability, we further selected four candidate genes as targets for residue detection: *LINC00428*, *LNCPRESS2*, *LINC00678* and *LOC105370482*.

### lncRNA detection assay based on ddPCR

**Figure 3:**
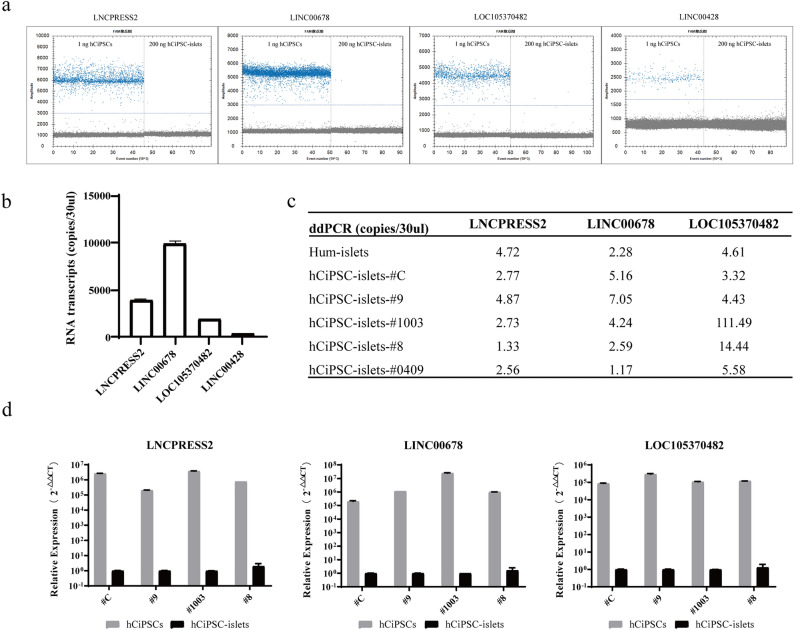
Detection the copy numbers using ddPCR and relatively expression using RT-PCR of selected markers. (**a**) The copy numbers of selected markers in 1 ng hCiPSCs RNA and 200 ng hCiPSC-islets RNA were measured by ddPCR. Each dot represented the fluorescence intensity of the droplet. The droplets can be divided into negative droplets and positive droplets according to their fluorescence amplitude. (**b**) The copy numbers of selected markers measured in the same input of hCiPSCs RNA. Results are presented as the mean ± standard deviation (n = 3). (**c**) RNA transcripts copy number of *LNCPRESS2*, *LINC00678* and *LOC105370482* in different samples, including human islets and five hCiPSC-islets. (**d**) Relative expression of *LNCPRESS2*, *LINC00678* and *LOC105370482* in four hCiPSCs lines, such as #C, #1003, #9 and #8, compared with hCiPSC-islets, respectively. Results are showed as the mean ± standard deviation (n = 3).

We used the more sensitive platform of ddPCR to test the capability of these four candidate markers for detecting residual undifferentiated cells. First, we measured the absolute RNA transcripts copies of each marker in hCiPSCs and hCiPSC-islets by ddPCR, using the same input of 1 ng of hCiPSCs RNA. The results showed that *LNCPRESS2*, *LINC00678*, *LOC105370482*, and *LINC00428* had average copy numbers of 3983.5, 9950, 939.5, and 392.2, respectively. On the other hand, these markers had negligible expression in 200 ng of hCiPSC-islets. This demonstrated a significant expression difference between these two cell types. *LNCPRESS2*, *LINC00678* and *LOC105370482* had high expression levels in hCiPSCs, which was consistent with the RT-qPCR results, but *LINC00428* had unexpectedly low expression (Figs. 3a, b). We re-designed the primers and probes for *LINC00428* to detect the shared sequences of its two alternative spliceosomes, but it did not improve the detection (data not shown). Therefore, we excluded *LINC00428* and selected *LNCPRESS2*, *LINC00678* and *LOC105370482* as the final promising markers after verification.

Next, we optimized the detection assay for the remaining markers to get better reaction performance. We designed orthogonal experiments to determine the optimal input volume of primer and probe in 30 μl ddPCR reaction system. The results showed that 3.6 μl of 10 μM primers and 1.5 μl of 10 μM probes were the best parameters, as they detected the highest copy numbers in all three reactions (Fig. S1a). We also tested different annealing temperatures from 56 °C to 62 °C: 56.6 °C, 58 °C, 60 °C and 62 °C. The results indicated that 56.6 °C was the optimal annealing temperature for all the assays (Fig. S1b). These optimization steps improved the efficiency and accuracy of our ddPCR assay for detecting residual undifferentiated cells.

### Expression level of lncRNAs in human islets and multi-hCiPSCs derived islets

To testify whether the selected lncRNAs could serve as universal biomarkers for detecting residual undifferentiated cells in hCiPSC-islet cells, we used the established ddPCR assay to measure the expression level of these lncRNAs in human islets and several hCiPSC lines-derived islets (Fig. 3c). We compared the expression level of *LNCPRESS2*, *LINC00678* and *LOC105370482* in human islets and hCiPSC-islets, evaluating their consistency and specificity. The result showed that the expression level of three lncRNAs was very low in human islets, with copy number of 4.72, 2.28, and 4.61 for *LNCPRESS2*, *LINC000678*, and *LOC105370482*, respectively. We used the detection results of human islets as a baseline since hCiPSC-derived islets were intended to mimic real human islets. For *LNCPRESS2* and *LINC000678*, the expression level of five hCiPSC lines-derived islets was similar to those of human islets, with consistent performance. However, for *LOC105370482*, the results of three hCiPSC lines-derived islets (9#, C#, 0409#) were similar to human islets, while 8# and 1003# derived islets showed higher results. This suggests that *LNCPRESS2* and *LINC00678* are more stable and specific markers than *LOC105370482* for different hCiPSC lines-derived islets. We also used RT-qPCR to measure the relative expression level of hCiPSCs and hCiPSCs-islets in four paired samples (Fig. 3d). Although the results varied between cell lines, we detected significant difference in all cases. These results demonstrated that the selected lncRNA markers were suitable for multi-hCiPSC derived islets, particularly for *LNCPRESS2* and *LINC000678*, making them promising candidates for universal biomarkers.

### Monitoring hCiPSC differentiation using lncRNA detection assay

**Figure 4:**
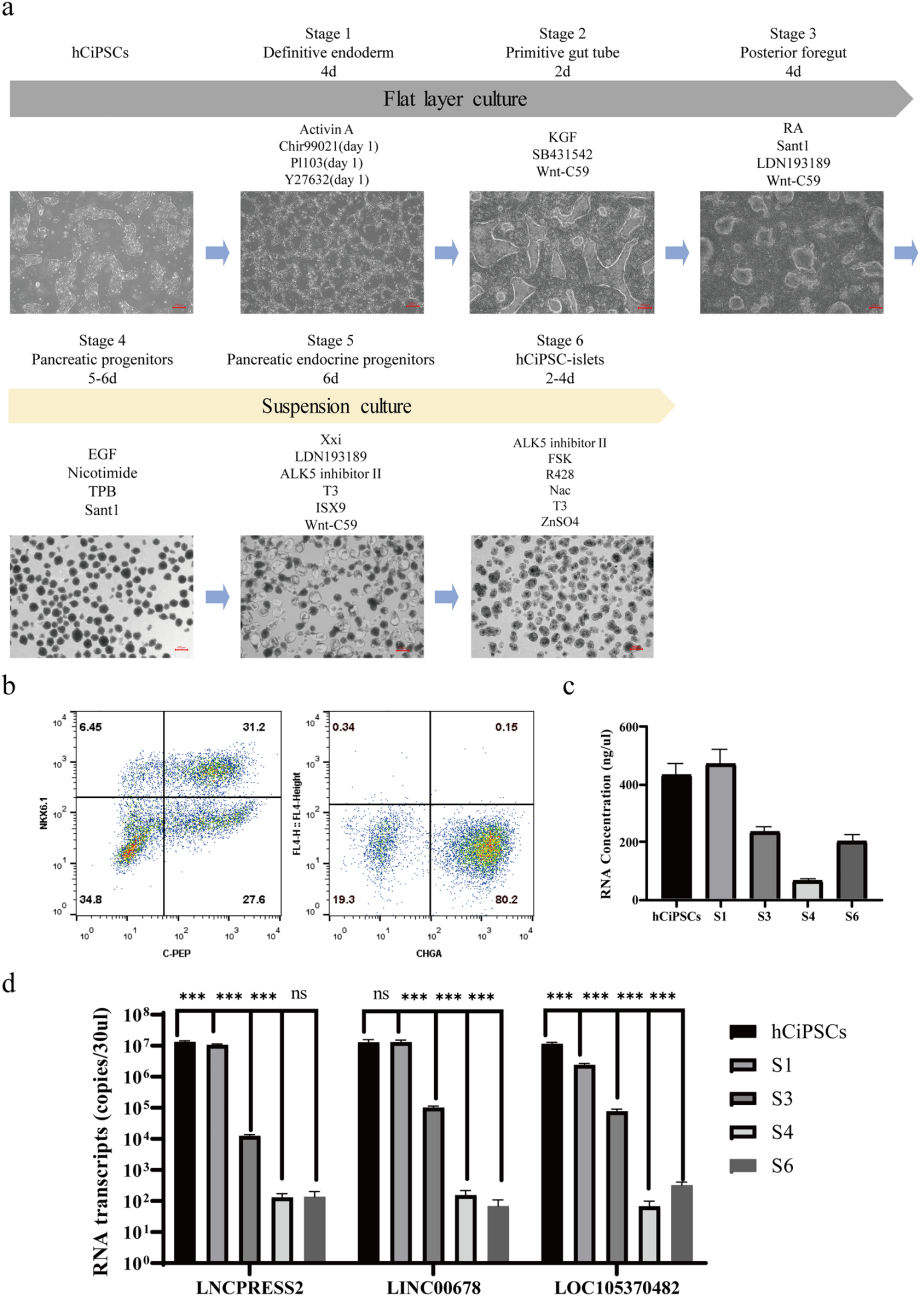
Monitoring hCiPSC-specific markers during hCiPSCs differentiated into islet cells. (**a**) Schematic of the hCiPSC-islets differentiation protocol. Representative images of cells at the end of each stage during hCiPSCs differentiation. Scale bar, 200 μm. (**b**) Flow cytometry analysis of the expression of β cell markers (C-peptide and NKX6-1) and endocrine marker (CHGA) in Stage 6 aggregates. (**c**) RNA concentration of cells at different stages. Results are presented as the mean ± standard deviation (n ≥ 5). (**d**) hCiPSC-specific markers expression at different stages. Results are presented as the mean ± standard deviation (n ≥ 7). *** p < 0.001, ns *p* > 0.05.

We measured the expression of these lncRNA markers during the differentiation of hCiPSCs to investigate their specificity. We used a six-stage protocol to differentiate hCiPSCs into islet cells in vitro, from definitive endoderm stage to final islets stage (Fig. 4a). We selected important stages for monitoring the expression of lncRNA markers, such as stage 1 for entering endoderm development, stage 3 for ending flat culturing, stage 4 for becoming pancreatic progenitors, and stage 6 for final cell products (Fig. 4a). We also monitored the differentiation process by observing the morphology and flow cytometry of markers specific for endocrine cells and beta cells. The results showed that the cells differentiated successfully into islet cells (Fig. 4a, b). We extracted total RNA from the same number of cells from hCiPSC, S1, S3, S4, and S6 stage cells, and measured the total RNA concentration and expression level of selected lncRNA markers (Fig. 4c, d). The concentration of total RNA varied with a decreasing trend along the differentiation process except for an increase at S6 stage (Fig. 4c). This might reflect the different RNA expression patterns of cells at different stages. The expression level of selected lncRNA markers also had a clear decreasing trend (Fig. 4d). For all three lncRNA markers, we observed drastic decreases in RNA transcripts expression at stage 3 and stage 4 with hundreds or thousands of fold change. Moreover, these lncRNA markers had their own characteristics during monitoring. For example, *LNCPRESS2* and *LOC105370482* decreased significantly at S1 stage but *LINC00678* did not. On the other hand, *LINC00678* continued to decrease at S6 with significant difference compared to S4, while *LNCPRESS2* remained unchanged and *LOC105370482* increased slightly at S6. These selected lncRNA markers were significantly differentially expressed between hCiPSC and its derived islets, which might be related to pluripotency. These results suggested that the lncRNA markers selected were consistent with the loss of pluripotency during the differentiation of hCiPSCs into terminal cells in vitro. Therefore, we proved that these three lncRNA makers were valuable for detecting residual undifferentiated cells.

### Cell spike-in study on lncRNA detection assay

**Figure 5:**
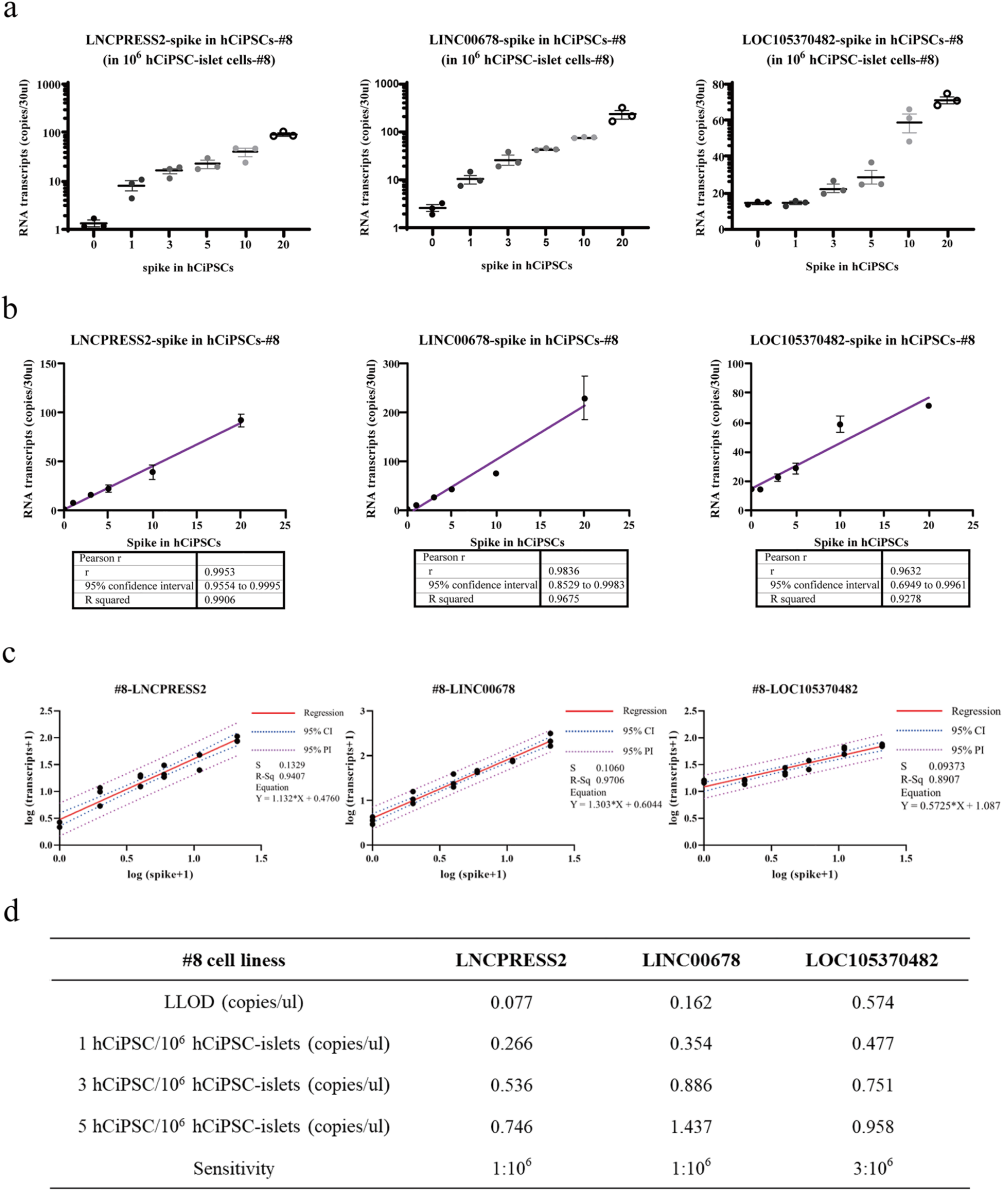
ddPCR detection results of selected marker in #8 cell spike-in samples. (**a**) Spike-in study to detection sensitivity of ddPCR-based method (for 0, 1, 3, 5, 10 and 20 hCiPSCs-#8 spiked in 10^6^ hCiPSC-islet cells-#8), n = 3. (**b**) Pearson's correlation analysis showed RNA transcripts of hCiPSC-specific markers were positively correlated with hCiPSCs spike-in numbers in #8 cell lines. Results are presented as the mean ± standard deviation (n = 3). (**c**) Linear regression analysis and curve fitting for *LNCPRESS2*, *LINC00678* and *LOC105370482* in #8 cell mixtures, n = 3. (**d**) Calculation of sensitivity of hCiPSC-specific markers in #8 cell lines.

We used cell spike-in samples to better assess the limit of detection (LOD) of the established ddPCR assays, as they were more realistic than RNA spike-in samples. We extracted total RNA from 0:10^6^, 1:10^6^, 3:10^6^, 5:10^6^, 10:10^6^, and 20:10^6^ spike-in samples, and synthesized cDNA for ddPCR assay. We conducted 13 technical replicates and 3 biological replicates per sample for each marker. The results showed that the copy numbers of marker-related transcripts increased with the increasing number of hCiPSCs for *LNCPRESS2* and *LINC00678*, but not for *LOC105370482* at 1: 10^6^ (Fig. 5a). *LNCPRESS2* and *LINC00678* could discriminate single hCiPSC, but *LOC105370482* could not. This suggests that *LNCPRESS2* and *LINC00678* are more sensitive markers than *LOC105370482* for detecting residual hCiPSCs.

We analyzed the results using correlation and linear regression model based on the copy numbers of the targets and the number of spiked-in hCiPSCs (Fig. 5b, c). The results showed that the copy numbers of RNA transcripts for hCiPSC-specific markers were positively correlated with the spike-in hCiPSCs cell numbers, with Pearson’s correlation coefficient of 0.99 (P < 0.0001) for *LNCPRESS2* in #8 cell line. We observed similar results for *LINC00678* and *LOC105370482* markers, with significant correlation coefficients of 0.97 (P = 0.0004) and 0.93 (P = 0.002), respectively (Fig. 5b). These high correlation coefficients indicate that the ddPCR assays are reliable and consistent for measuring the expression levels of these markers. In order to verify the capability of these markers for other hCiPSC line, we also performed the same study on another hCiPSC line #0409, and obtained similar results (Figs. S2a, b). A linear regression analysis showed that *LNCPRESS2*, *LINC00678* and *LOC105370482* had good coefficients of determination: 0.94, 0.97 and 0.89 in #8 cell line, respectively (Fig. 5c). We found similar results in #0409 cell line with the coefficients of determination of 0.91, 0.93 and 0.90, respectively (Fig. S2c). In both cell lines, these markers showed similar detection capabilities. These results demonstrated that these lncRNA markers were promising in different hCiPSC lines and their derived islets.

We calculated the LLOD (the lower limit of detection, LLOD = mean + 3.3*SD^33^) of each selected marker, using hCiPSC-islets samples as the negative control (Fig. 5d, Fig. S2d). The results showed that lncRNA assay could detect as few as 1, 1 and 3 undifferentiated hCiPSCs in the background of 10^6^ hCiPSC-derived islet cells for *LNCPRESS2*, *LINC00678* and *LOC105370482*, respectively. This means that *LNCPRESS2* and *LINC00678* could quantify 0.0001% hCiPSCs in 10^6^ hCiPSC-derived islet cells, and *LOC105370482* 0.0003%. The expression of these lncRNA in single or 3 hCiPSCs could be calculated and used for comparing with that of islet cells, which could determine whether the islet cells containing 1 or 3 hCiPSCs or not.

In this study, based on the fact that copy number of lncRNA transcrispts of both 10^6^ of hCiPSC line #8 and #0409 derived islets products were less than a single hCiPSC, we concluded that the residual undifferentiated cells were less than 0.0001% using the lncRNA assay of *LNCPRESS2* and *LINC00678* as biomarkers. This method can help ensure the safety and quality of hCiPSC-derived islet cells for clinical applications.

## Discussion

The aim of this study was to establish a lncRNA detection assay based on ddPCR platform to detect residual undifferentiated cells in hCiPSC-islet cells. We selected three lncRNA marker, *LNCPRESS2*, *LINC00678* and *LOC105370482* that were significantly differentially expressed between hCiPSCs and hCiPSC-islets, with high expression in hCiPSCs and low expression in hCiPSC-islets. We also observed that the expression levels of these lncRNA markers gradually decreased during hCiPSC differentiation in vitro. We demonstrated that our assay could accurately measure the RNA transcript copy numbers of these lncRNA markers in cell spike-in samples, which had a positive correlation with hCiPSC cell quantities. Moreover, we showed that our assay could detect as few as 1, 1 and 3 undifferentiated hCiPSCs in the background of 10^6^ hCiPSC-derived islet cells for *LNCPRESS2*, *LINC00678* and *LOC105370482*, respectively. This indicates that our assay has a high sensitivity and specificity for quantifying the residual number of hCiPSCs in their derived islet cells. Furthermore, we could estimate whether residual undifferentiated cells were above or below 0.0001% in derived islets product by using *LNCPRESS2* and *LINC00678* as biomarkers. These results suggest that our lncRNA detection assay based on ddPCR is a reliable and robust tool for assessing the safety and quality of hCiPSC-derived islet cells. This method can help ensure that the residual undifferentiated cells are below the acceptable threshold for clinical applications.

Cell therapy using iPSCs is a promising approach to treat various diseases because of their unique properties. They can be reprogrammed from the patient’s own cells, avoiding ethical issues, and they have the ability to self-renew and differentiate into any cell type. So far, many hiPSC-derived cell products have entered the scope of clinical research, such as retinal pigment epithelium (RPE) cells, cardiomyocytes, chondrocytes, neural stem/progenitor cells, natural killer cells, and islet cells^34,35^. Among these products, islet cells are especially relevant for diabetes treatment, as they can produce insulin and regulate blood glucose levels. Du et al. showed that islets derived from hCiPSCs were able to relieve hyperglycemia and improve overall glycemic control in a pre-clinical non-human primates experiment. This released a strong confidence in stem-cell-derived islets in clinical research for diabetes treatment. However, not all iPSCs are equally safe and efficient for generating islet cells. CiPSCs are safer than OSKM-iPSCs (iPSCs induced by *OCT4*, *SOX2*, *KLF4*, and *c-Myc*) according to the chimera experiments that the chimeric mice generated from CiPSCs were 100% viable and healthy for up to 6 months ^5^. However, the risk of teratoma formation or tumorigenesis is always a great concern in such clinical trials, especially considering immune system is suppressed in these patients. Therefore, it is essential to develop reliable methods to detect any residual undifferentiated cells in the derived islet cells before transplantation.

We screened two groups of biomarkers from RNA-seq data: protein-coding RNA (*LDHA*, *TDGF1*, and *TUBB*) and non-coding RNA (*ESRG*, *LINC00678*, *LOC105370482*, *LINC00428*, and *LNCPRESS2*). We also included several protein-coding RNA, such as *NANOG* and *OCT4*^36^, that are crucial for manipulating pluripotent network and theoretical specific for iPSCs. However, we found that these protein-coding RNA markers were not suitable for detecting residual undifferentiated cells in hCiPSCs derived islets product by RT-qPCR. The difference of relative expression between hCiPSCs and derived islets was not large enough to discriminate minor changes. Moreover, the specificity of biomarkers was determined by the iPSC derived cells. For example, *LIN28A*, which was reported as a biomarker to detect residual undifferentiated iPSCs with a sensitivity of 0.001% in primary cardiomyocytes and 0.002% in RPE cells^37,38^, was not effective for detecting residues in hepatocyte. Therefore, we excluded the protein-coding RNA markers from further study. On the other hand, we found that non-coding RNA showed better performance. Previous studies reported that *ESRG* was a robust marker for detecting residuals amongst differentiated cells from three germ lineages^21^ and a universal marker to detect hiPSCs residues in different types of hiPSC-derived cells^39^. However, our results demonstrated that *ESRG* was not specific enough for hCiPSC-islet cells. Instead, we indentified *LNCPRESS2*, *LINC00678* and *LOC105370482* as valuable lncRNA markers for detecting residual hCiPSCs in derived islets. These markers were suitable across several reprogrammed hCiPSC lines, making them universal markers for derived islets. Several studies have demonstrated that specific lncRNAs are essential for maintaining pluripotency and interacting with core TFs OCT4, NANOG, and SOX2 in mESC^27^. For example, *LNCPRESS2* maintain pluripotency through coordinating with pluripotency-specific genes^40^. *LOC105370482* is highly expressed in placenta and might be related with cell differentiation^41^. In our study, we observed that the expression level of these lncRNAs decreased along with the differentiation process, indicating these lncRNAs may have similar roles in hCiPSCs. Further study should be done to verify the function of these lncRNAs using technologies such as gene knockdown in order to elucidate the relationship with pluripotency.

Besides the RNA transcripts discovered and verified above, we also found some microRNA (miRNA) differential expressed. Chung et al reported an iPSC-specific miRNA assay for detection of residual undifferentiated cells in natural killer cells, which indicate that miRNA might be a promising biomarker^22^. However, we could not achieve a satisfactory sensitivity using miRNA as a biomarker for hCiPSC-islet cells. We established a stem-loop RT-qPCR assay for miRNA and got a detection of LOD of 0.05%. In order to improve the sensitivity, we built an assay based on ddPCR platform using TaqMan probe and found that the expression of target miRNAs in hCiPSC-islets was too high to discriminate subtle transcripts of hCiPSCs (data not shown). We also tried to isolate miRNAs from cell culture supernatant which was showed to be an efficient method for detecting miRNAs^42,43^, but still could not reach enough detection sensitivity. This illustrates the pivotal role in selecting a proper biomarker for detecting specific iPSC derived cell product.

To our knowledge, it is the first report on assessing the residual hCiPSCs in derived islet cells. Our study discovered hCiPSCs specific lncRNA markers hardly expressed in its derived islet cells, with *LOC105370482* reported for the first time. Our lncRNA detection assay could reach 0.0001% sensitivity which among the top performance of research so far (Table 1) and make sure that #0409 and #8 hCiPSC line derived islets product containing residual undifferentiated cells less than 0.0001%. This suggests that our lncRNA detection assay based on ddPCR is a reliable and robust tool for quantifying the residual number of hCiPSCs in its derived islet cells. On the other hand, this study also has some limitations. First, the RNA-seq data set is relatively small, might not cover enough hCiPSC lines and their derived islets. Second, other genetic factors should be considered besides RNA expression, such as gene methylation and RNA splicing, in order to improve overall detection performance. Third, we also should verify the general applicability of these three markers in our future research and see which hCiPSC-derived cell types they can be used for. Besides undifferentiated hCiPSCs, the cell types and proportions are also affecting the quality of hCiPSC-islets. Future work will be conducted on establishing detection systems for different cell types on the digital PCR platform using new biomarkers, which can be used to monitor the proportions of various cells after each batch of differentiation. All in all, the lncRNA detection assay in this study provides inspiration for detecting other cell products derived from hCiPSCs.

**Table 1:** Residual undifferentiated cells detection assays based on qPCR or ddPCR.

Platform	Reprogramming method	hiPSC-derived cell	biomarkers	Sensitivity	References
qPCR	Lentiviral reprogramming	RPE cells	LIN28A	0.002%	^16^
ddPCR	Lentiviral reprogramming	cardiomyocytes	LIN28A	0.001%	^39^
ddPCR	Lentiviral reprogramming	neural progenitor cells	OCT4, TDGF1 and LIN28A	0.002%	^37^
qPCR	Lentiviral reprogramming	Hepatocytes	ESRG, SFRP2 and CNMD	0.005%, 0.025% and 0.025%,	^21^
ddPCR	Episomal Reprogramming	Natural killer cells	miR-302a-5p, miR-302c-3p and miR-302d-5p	0.0005%, 0.0003% and 0.001%	^22^
qPCR	Lentiviral reprogramming	neural progenitor cells supernatant	miR-302b	0.01–0.001%	^43^
ddPCR	Episomal Reprogramming	Universal	ESRG and ZSCAN10	0.0001%	^40^
qPCR	Lentiviral reprogramming	cardiomyocytes	ESRG, LINC00678, CAMKV, IDO1, CNMD, L1DT1, LCK, VRTN and ZSCAN10	0.001% to 0.1%	^44^
ddPCR	Chemical reprogramming	islets	LNCPRESS2, LINC00678 and LOC105370482	0.0001%	This study

## Methods

### Cells used in this study

All cells used in this study was listed in Table S1. These cells were generated and cryopreserved before this study. Specifically, 12 hCiPSC cell lines were used, with 7 for biomarker discovery and 5 for biomarker verification. Additionally, 13 hCiPSC-islets were used, with 8 for biomarkers discovery and 5 for biomarkers verification. When used, these cells were thawed in a 37 °C water bath, centrifuged at 350*g* for 3 min, and resuspended in different media. hCiPSC-islets were resuspended in DMEM-basic with 1% B27 (Gibco, 12587–010), while hCiPSCs were in mTeSR1 (STEMCELL Technologies, 85850). hCiPSCs were further dissociated into single cells with Accutase (EMD Millipore, SCR005) and both type of cells was counted with Countess II Automated Cell Counter (Invitrogen, AMQAX1000).

hCiPSCs were further differentiated into islets using a modified six-stage protocol^8^. Cryopreserved hCiPSCs were recovered and cultured on Matrigel-coated dishes (BD BioSciences, 356231, 1:40 diluted) in mTeSR1 medium with 5% CO_2_ at 37 °C. hCiPSCs were then differentiated in vitro. Cells at stage1, 3, and 4 were partially harvested and stage 6 was fully harvested for analysis their RNA expression of target markers.

### In vitro differentiation to generate hCiPSC-islets

The protocol was followed Du’s work^8^. Briefly, the medium formulation for each stage was as below: Stage 1 (4 d). For day 1, MCDB131 (Gibco, 10372–019) supplemented with glucose, GlutaMAX (Gibco, 35050–061), Pen/Strep, B27, activin A, vitamin C, Chir99021, PI103 and Y27632. For days 2–4, culture medium was refreshed every day in MCDB131 with glucose, GlutaMAX, Pen/Strep, B27, activin A and vitamin C. Stage 2 (2 d). MCDB131 supplemented with glucose, GlutaMAX, Pen/Strep, BSA (Sigma-Aldrich, A4612), KGF, vitamin C, SB431542 and Wnt-C59. Stage 3 (4 d). DMEM-basic (Gibco, C11965500BT) supplemented with B27, Pen/Strep, retinoic acid, LDN193189, Sant1 and Wnt-C59. At the end of Stage 3, the cells were dispersed and seeded in AggreWell Microwell Plates (STEMCELL Technologies, 27940) in Stage 4 medium with Y27632 for 20 h and then transferred into an ultra-low attachment six-well plate (Beaver Bio, 40406) with stage 3 medium. Stage 4 (5–6 d). DMEM-basic supplemented with GlutaMAX, B27, Pen/Strep, EGF, TPB, nicotinamide, Sant1 and vitamin C. Stage 5 (6 d). DMEM-basic supplemented with Pen/Strep, GlutaMAX, B27, ALK5 inhibitor II, LDN193189, T3, ISX9 (3–6 d), heparin, γ-secretase inhibitor Xxi, Wnt-C59, Y27632 and vitamin C. Stage 6 (2–4 d). DMEM-basic supplemented with B27, Pen/Strep, ALK5 inhibitor II, R428, T3, forskolin, heparin, zinc sulfate, N-Acetyl-ʟ-cysteine and vitamin C.

### RNA extraction and library preparation for RNA-seq

Total RNA was isolated using Direct-zol RNA MiniPrep Kit (Zymo Research, R2053) following manufacturer’s protocol. The concentration of total RNA was measured using Nanodrop one (Thermo Scientific, 701–058112) and 1 μg of total RNA was used for RNA-seq libraries constructed using NEBNext Ultra RNA Library Prep Kit for Illumina (NEB England BioLabs, E7775) following manufacturer’s instruction. Briefly, rRNA was depleted from total RNA through probe hybridization method, and then fragmented and transcribed into cDNA. Through end repair/dA-tailing, adaptor ligation and PCR, the final libraries were quality controlled using Qubit 4.0 (Thermo Scientific, Q33238) and 4150 TapeStation (Agilent Technologies, G2992AA) for library concentration and size distribution, respectively. The libraries were sequenced using the Illumina HiSeq X Ten system using 2 × 150 bp paired-end sequencing strategy with 8 G bytes per cell.

### RNA-seq data analysis

Quality control was performed using FastQC (version 0.11.8) for all libraries of seven iPSCs cells and eight hCiPSC-islets. The raw RNA-seq FASTQ were trimmed using TrimGalore (version 0.6.10). Mapped the clean reads to the human reference genome GRCh38.84 using HISAT2 (version 2.2.1). The number of reads mapped to each gene was counted using the featureCounts (version 2.0.3). The average TPM of each gene in hCiPSCs and hCiPSC-islets was calculated, and then used hCiPSC-islets as control, hCiPSCs as experiment to calculate the foldchange. Heatmap and volcano plot were generated using the ComplexHeatmap (version 2.14.0) and ggplot2 (version 3.4.0), respectively. The color of heatmap is drawn by the TPM value, each bar represents the expression level of each gene. Volcano plot showing differential expression genes between hCiPSCs and hCiPSC-islets.

### Spike-in study

Spike-in studies, including RNA spike-in study and cell spike-in study, were performed to evaluate the sensitivity and performance of potential biomarkers.

For RNA spike-in study, total RNA of hCiPSCs and hCiPSC-islets with different amounts were mixed to generate different percentages of samples. For example, 10% spike-in sample means mixing 1.8 μg of hCiPSC-islets RNA with 0.2 μg of hCiPSCs RNA, and 1% means mixing 1.98 μg of hCiPSC-islets RNA with 0.02 μg of hCiPSCs RNA. It was similar for 0.1%, 0.01%, 0.001%, and 0% samples used in RT-qPCR assay.

For cell spike-in study, hCiPSCs were stained with trypan blue and picked up cell one by one using an in-house capillary needle connected to a pipette under the microscope. hCiPSC with intact cell membranes and no trypan blue uptake was selected and spiked them into 10^6^ hCiPSC-islet cells that were pre-counted using Countess II Automated Cell Counter. 0:10^6^, 1:10^6^, 3:10^6^, 5:10^6^, 10:10^6^, and 20:10^6^ spike-in samples were prepared by adding 0, 1, 3, 5, 10 and 20 hCiPSCs respectively.

### RT-qPCR

Total RNA was extracted using the MiPure cell/Tissue miRNA Kit (Vazyme, RC201) and its concentration was measured using Nanodrop. 2 μg of all total RNA (hCiPSCs, hCiPSC-islets and RNA spike-in sample) was taken to synthesize cDNA using Evo M-MLV RT Mix Kit with gDNA Clean (AG, 11728) in 20 μl following manufacturer’s protocol. 1 μL of cDNA was further taken into quantitative PCR performed using SYBR Green Premix Pro Taq HS qPCR Kit (AG, 11718) and designed primers (Supplementary Table 2) on 7500 Real Time PCR system (Applied Biosystems) at a final volume of 20 μL/well. Relative expression levels of each gene were normalized to the housekeeping gene ACTB. The results were analyzed using the ΔΔCt method.

### ddPCR

Total RNA was extracted from individual cell sample or cell spike-in sample. 10 ng hCiPSCs RNA, 2 μg hCiPSC-islets RNA and 2 μg spike-in RNA were taken to synthesize cDNA in 20 μl, respectively. 2 μL cDNA was added into PCR reaction mixture together with 15 μl ddPCR supermix for probes with UNG (TargetingOne, 23003), primer set and probe (Supplementary Table 3) in a final volume of 30 μL. Each ddPCR mixture was then loaded into the 8-well plate to generate droplets into 8-well PCR tube strip according to the manufacturer’s protocol. The droplets contained PCR tube strip was then placed in the thermal cycler and followed the PCR condition: 95 °C for 10 min, then 40 cycles of 94 °C for 30 s and 60 °C for 1 min (ramping rate changed to 1.5 °C/s), and a final at 12 °C for 5 min. For cell spike-in study, total RNA was used to reverse-transcribed with volume of 40 μL. Each ddPCR reaction contained 3 μL cDNA, each samples performed 13 technical replicates and 3 biological replicates. ddPCR was performed in optimized condition (Fig. S1). The PCR products were subjected to droplets analysis according to manufacturer’s protocol. Additionally, no-template control (NTC) was added in each batch. Results analysis was conducted in GraphPad Prism 8.

### Supplementary Information

Supplementary Information.

## Data Availability

The processed data are provided in the Figures and Tables. Additional data requests can be made to the corresponding author (yue.pu@reprogenix.com).
