# Supplementary material for: Estimating residual undifferentiated cells in human chemically induced pluripotent stem cell derived islets using lncRNA as biomarkers
Source: Sci Rep. 2023 Sep 30;13:16435. doi: 10.1038/s41598-023-43798-0 (PMC10542758; doi:10.1038/s41598-023-43798-0)
Supplement: Supplementary file 1 — Supplementary Information. [file 41598_2023_43798_MOESM1_ESM.pdf]

Supplemental Information

Estimating residual undifferentiated cells in human chemically induced pluripotent stem cell derived islets using lncRNA as biomarkers

Yandan Wu, Zhenzhen Zhang, Shuangshuang Wu, Zhaolong Chen, Yue Pu

Table S1. Cell sources in this study.

Number	Sample ID	Origination	Donor	Purpose
1	hCiPSCs-1	hADSCs	Donor 1	
2	hCiPSCs-2	hADSCs	Donor 2	
3	hCiPSCs-3	hADSCs	Donor 2	
4	hCiPSCs-4	hADSCs	Donor 3	RNA-seq for biomarker discovery
5	hCiPSCs-5	hADSCs	Donor 3	
6	hCiPSCs-6	hADSCs	Donor 4	
7	hCiPSCs-7	hADSCs	Donor 5	
8	hCiPSCs-9#	hADSCs	Donor 6	
9	hCiPSCs-C#	hADSCs	Donor 7	
10	hCiPSCs-1003#	hADSCs	Donor 8	RT-qPCR and ddPCR for biomarker verification
11	hCiPSCs-8#	hADSCs	Donor 9	
12	hCiPSCs-0409#	hADSCs	Donor 10	
13	hCiPSC-islets-1	hCiPSCs-1	Donor 1	
14	hCiPSC-islets-2	hCiPSCs-1	Donor 1	
15	hCiPSC-islets-3	hCiPSCs-2	Donor 2	
16	hCiPSC-islets-4	hCiPSCs-3	Donor 2	RNA-seq for biomarker discovery
17	hCiPSC-islets-5	hCiPSCs-4	Donor 3	
18	hCiPSC-islets-6	hCiPSCs-5	Donor 3	
19	hCiPSC-islets-7	hCiPSCs-6	Donor 4	
20	hCiPSC-islets-8	hCiPSCs-6	Donor 4	
21	hCiPSCs-islets-9#	hCiPSCs-9#	Donor 6	
22	hCiPSCs-islets-C#	hCiPSCs-C#	Donor 7	
23	hCiPSCs-islets-1003#	hCiPSCs-1003#	Donor 8	RT-qPCR and ddPCR for biomarker verification
24	hCiPSCs-islets-8#	hCiPSCs-8#	Donor 9	
25	hCiPSCs-islets-0409#	hCiPSCs-8#	Donor 10	

hADSCs: human adult adipose-derived mesenchymal stromal cells.

Table S2. primer sequences for RT-qPCR.

Gene	Primer sequence 5’-3’
ESRG-F	GACTCCTTCTTGGCTTACTG
ESRG-R	TGTGTGAGCAACAAGGCTGT
POU5F1-F	AAACCCACACTGCAGCAGAT
POU5F1-R	TGCATAGTCGCTGCTTGATC
NANOG-F	CCATCCTTGCAAATGTCTTC
NANOG-R	CCATCCTTGCAAATGTCTTC
CNMD-F	TCCGACAAAGTTCCCATTGC
CNMD-R	GCACAGCTCCCGAAATGAG
LDHA-F	TATTGGAAGCGGTTGCAATC
LDHA-R	ACACCAGCAACATTCATTCC
TUBB-F	TCTGTTCGCTCAGGTCCTTT
TUBB-R	TACCACATCCAGGACAGAAT
TDGF1-F	AGTCTTTGAACTGGGATTAG
TDGF1-R	CATCTCTGAAGGCCAGGTAT
LINC00428-F	GACCAGCCCAAGAAACATCT
LINC00428-R	CATCAGCAGTCCCTGTTAG
LNCPRESS2-F	AACCAGCCCAAGGAACATCT
LNCPRESS2-R	CCTTCATCACCTTGGAGCTT
LOC105370482-F	AAGAAATCACCTCGGATGTC
LOC105370482-R	CCTGTCGACAGACATTTAGG
LINC00678-F	CCAACGAACATCTCACCAAT
LINC00678-R	TCCCGTCATTCTGCTAACAC
ACTB-F	GAGCTGCGTGTGGCT
ACTB-R	GCACAGCCTGGATAGCAAC

Table S3. primer and probe sequences for ddPCR.

Gene	Primer and probe sequence 5’-3’
LINC00428-F	CTTCGGGTAACTCTAACAGT
LINC00428-R	GCCCCAGATATAGGATTCAT
LINC00428-P	5’-FAM-AGTCCCTGTTAGCCTGGCCT-MGB-3’
LNCPRESS2-F	AACCAGCCCAAGGAACATCT
LNCPRESS2-R	CCTTCATCACCTTGGAGCTT
LNCPRESS2-P	5’-FAM-AGTGCTGAATCTCAGGTACC-MGB-3’
LOC105370482-F	AAGAAATCACCTCGGATGTC
LOC105370482-R	CCTGTCGACAGACATTTAGG
LOC105370482-P	5’-FAM-CGGCTGTCTCATGACTACAA-MGB-3’
LINC00678-F	CCAACGAACATCTCACCAAT
LINC00678-R	TCCCGTCATTCTGCTAACAC
LINC00678-P	5’-FAM-CCTGCTCATACTTGATCTGG-MGB-3’

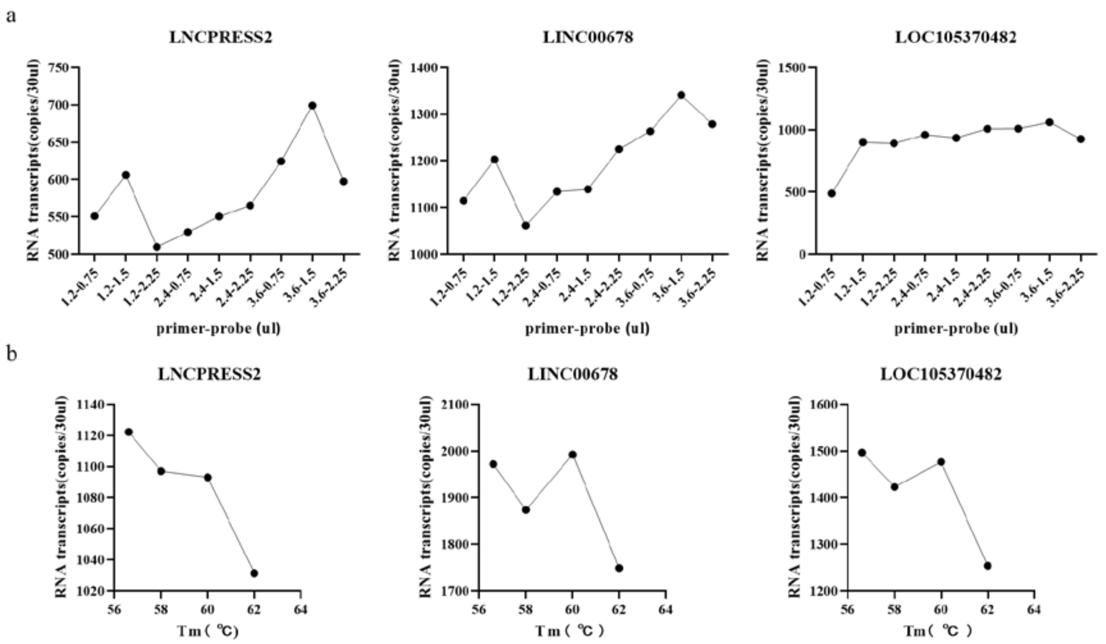

Figure S1. ddPCR detection system optimization. (a) The optimal ratio of primer and probe concentrations were explored. (b) The gradient tests for four annealing temperatures were performed from 56°C to 62°C.

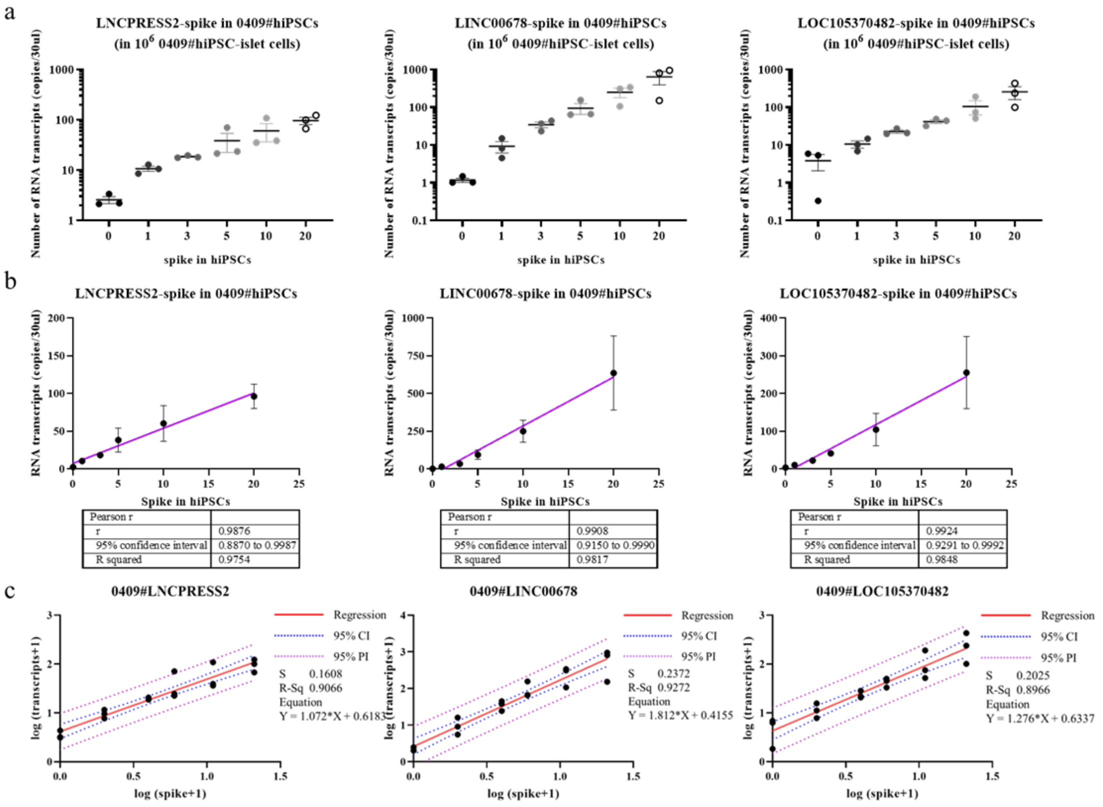

d

#0409 cell lines	LNCPRESS2	LINC00678	LOC105370482
LLOD (copies/ul)	0.162	0.234	0.436
1 hCiPSC/10^6 hCiPSC-islets (copies/ul)	0.287	0.517	0.352
3 hCiPSC/10^6 hCiPSC-islets (copies/ul)	0.614	1.152	0.752
5 hCiPSC/10^6 hCiPSC-islets (copies/ul)	1.280	3.149	1.388
Sensitivity	1:10^6	1:10^6	3:10^6

Figure S2. ddPCR detection results of selected marker in #0409 cell mixtures. (a) Spike-in study to detection sensitivity of ddPCR-based method (for 0, 1, 3, 5, 10 and 20 hCiPSCs-#0409 spiked in 10^6 hCiPSC-islet cells-#0409), n=3. (b) Pearson's correlation analysis showed positive correlations between the RNA transcripts of markers and hCiPSCs-#0409 spike-in numbers in hCiPSC-islets-#0409. Results are presented as the mean ± standard deviation (n=3). (c) Linear regression analysis and curve fitting for LNCPRESS2, LINC00678 and LOC105370482 in #0409 cell mixtures, n=3. (d) Sensitivity of hCiPSC-specific markers in #0409 cell lines.
